# The German version of Child Perceptions Questionnaire for children aged 8 to 10 years (CPQ-G8–10): translation, reliability, and validity

**DOI:** 10.1007/s00784-020-03451-w

**Published:** 2020-07-14

**Authors:** Katrin Bekes, Markus Ebel, Maisa Omara, Sarra Boukhobza, Nicoletta Dumitrescu, Julia Priller, Nejra Kulovic Redzic, Anuscha Nidetzky, Tanja Stamm

**Affiliations:** 1 Department of Paediatric Dentistry, University Clinic of Dentistry, Medical University of Vienna, Sensengasse 2a, 1090 Vienna, Austria; 2 Private practice, Bergisch Gladbach, Germany; 3 Center for Medical Statistics, Informatics, and Intelligent Systems, Section for Outcomes Research, Medical University of Vienna, Spitalgasse 23, 1090 Vienna, Austria

**Keywords:** Oral health-related quality of life (OHRQoL), Child Perceptions Questionnaire (CPQ), Psychometric properties, Reliability, Validity

## Abstract

**Objectives:**

The aims of this study were to develop a German version of the Child Perceptions Questionnaire for children aged 8 to 10 years (CPQ-G8–10), a measure of oral health-related quality of life, and to assess the instrument’s reliability and validity.

**Methods:**

The original English version of the CPQ8–10 questionnaire was translated into German (CPQ-G8–10) by a forward-backward translation method. A total of 409 8- to 10-year-old children who were recruited at the Department of Paediatric Dentistry in Vienna, Austria, participated in this study. The children self-completed the CPQ-G8–10 and were clinically examined for the presence of dental caries and plaque accumulation. Reliability of CPQ-G8–10 was investigated in a subsample of 58 children after 3 weeks.

**Results:**

Questionnaire summary score test-retest reliability was 0.85 (intraclass correlation coefficient, 95% confidence interval (CI) ranging from 0.75 to 0.91) and internal consistency was 0.88 (Cronbach’s alpha, lower limit of the 95% CI: 0.87). Validity of the CPQ-G8–10 questionnaire was supported by correlation coefficients with global ratings of oral health of − 0.40 (95% CI − 0.49 to − 0.31) and overall well-being of − 0.26 (95% CI − 0.33 to − 0.13) which met the expectations. Mean CPQ-G8–10 scores were statistically significantly higher in children with caries (dmft+DMFT > 0) compared with caries-free children (*p* = 0.02).

**Conclusions:**

The German version of the CPQ8–10 was found to be reliable and valid in children aged 8 to 10 years.

**Clinical relevance:**

These findings enable assessments of oral health-related quality of life in German speaking 8- to 10-year-old children.

**Electronic supplementary material:**

The online version of this article (10.1007/s00784-020-03451-w) contains supplementary material, which is available to authorized users.

## Introduction

Currently, there is a growing move in pediatric dentistry towards the use of patient-focused assessments as clinical indicators alone do not reveal the full impact of oral conditions on the psychosocial well-being of a patient [1, 2]. Thus, the concept of oral health-related quality of life (OHRQoL) has also become an important measure to assess oral health status in children. Specific issues could arise when measuring OHRQoL in younger patients due to their phase of physical cognitive, emotional, social, and language development, as oral health and health cognition are considered age-dependent [3, 4]. Several instruments have been specifically developed for specific age groups. The most often used ones include the Early Childhood Oral Health Impact Scale (ECOHIS) for pre-school children [5], the Child Perceptions Questionnaire [6, 7], the Child Oral Health Impact Profile (COHIP) [8], and the Child Oral Impacts on Daily Performances (C-OIDP) [9]. For children aged 8 to 10 years, the Child Perceptions Questionnaire (CPQ8–10) is frequently used. Several studies show the process of translation, cultural adaptation, and validation of this measure for different countries and cultures making the questionnaire a promising tool for international collaboration [10–15]. However, a German version did not exist to date.

As the Medical Outcome Trust has made the existence of such instrument versions a criterion for the assessment and review of health-related quality of life instruments [16], the aims of our study were to develop a German version of the Child Perceptions Questionnaire for children aged 8 to 10 years and to assess the instrument’s reliability and validity.

## Materials and methods

### Development of the German version of the CPQ8–10

The English language self-complete Child Perceptions Questionnaire to determine the frequency of various oral health-related impacts in 8–10-year-olds (CPQ8–10) was developed and validated in Toronto, Canada [7]. The CPQ8–10 contains a total of 25 items subdivided into four domains: five items on oral symptoms (items 1 to 5), five questions on functional limitations (items 6 to 10), five questions on emotional well-being (items 10 to 15), and ten questions on social well- being (items 16 to 25). Questions ask about the frequency of events, e.g., symptoms such as pain or bad breath in the child’s last 4 weeks. Responses are made on an ordinal scale (0 = never, 1 = once/twice, 2 = sometimes, 3 = often, 4 = every day/almost every day). Higher scores refer to a worse OHRQoL status. Summing the response codes for the questionnaire items generates domain scores/subscales and an overall CPQ8–10 score. Due to the psychometric nature of our analysis, e.g., assessing the reliability by comparing accordance between answers of two time points, we decided not to impute missing items and the analyses were performed with complete observations only. The instrument’s summary score ranges from 0 to 100. A summary score of zero indicates the absence of any problems, and higher CPQ scores represent more impaired OHRQoL. In addition to the 25 items, the CPQ-8–10 includes two questions asking the child for a global rating of the oral health and the overall well-being. These global ratings had a five-point response format (excellent, very good, good, moderate, poor).

Following accepted standards for the cross-cultural adaptation of health-related quality of life questionnaires [17], the original 25 English-language items were translated into German. Two independent native German speakers with extensive knowledge of English language and experience in translating health-related questionnaires carried out two independent translations. Both translations were merged into one version. This version was back-translated into English by two native English speakers. Finally, these two versions were synthesized and the final questionnaire (CPQ-G8–10) was compared with the original English-language instrument. A committee consisting of the two forward translators, the two back-translators, and a methodologist supervised the entire translation process.

### Subjects and setting

Children for this study were recruited from the Department of Paediatric Dentistry at the Medical University of Vienna, Vienna, Austria. To be included in the study, the children had to be 8 to 10 years of age and German speaking. Furthermore, no underlying serious medical conditions and no take-in of long-term medication were required. The children were invited to complete the CPQ-G8–10. Those children who attended the clinic for a second time within 3 weeks for prophylaxis were invited to complete the CPQ-G8–10 a second time.

The children were clinically examined by one dentist who was trained and calibrated in accordance with the WHO Basic Methods criteria for visual assessment of dental caries [18]. The dmft/DMFT (sum of decayed, missing, and filled teeth in the permanent dentition) index and its components were used to assess caries status. Dental plaque accumulation was evaluated using the simplified additive index for plaque accumulation described by Ambjørnsen et al. (score “0” = no visible plaque, score “1” = visible plaque (per child)) [19]. This index was chosen to be comparable and consistent with the study design of the development of the German version of the CPQ11–14 [1].

At the time of enrollment in the study, parents signed an informed consent form before a child’s verbal assent was sought. A child’s dissent superseded the parental consent. When a child’s verbal assent was obtained, the assent was documented. Approval for this study was obtained from the ethics committee of the local University Review Board (Medical University of Vienna; #2015–2025).

### Reliability assessment

We analyzed the impact sections and the instruments whole summary scores’ internal consistency and temporal stability. To determine internal consistency, we calculated Cronbach’s alpha [20]. According to guidelines [21], Cronbach’s alpha values of 0.70–0.80 were considered “satisfactory” for a reliable comparison between groups.

To determine the temporal stability of scores, test-retest reliability was assessed in a convenience sample of children. The interval between the first test interview and the retest interview was 3 weeks. The test-retest reliability was measured by calculating intraclass correlation coefficients (ICC). The calculation of the ICCs was based on a two-way mixed effects model because the two consecutive measurements (test-retest setting) were not randomized samples and we assumed that there should be absolute agreement between the measurements of the two consecutive time points [22–24]. According to guidelines [25], reliability coefficients > 0.75 are considered excellent. For sample size calculation, we estimated that 51 individuals were required for detecting an ICC of 0.8 with a desired confidence interval width of 0.2 using the ICC package in R (www.r-project.org).

### Validity assessment

To ensure that the instrument measured what it is supposed to measure, score validation was performed. The approach taken by the developers of the original CPQ8–10 and those of other language versions was followed to be compatible with previous validation efforts. Therefore, we determined construct validity by computing Spearman rank correlations of the children’s reported scores for the total scale and each domain with the global rating of oral health and overall well-being (excellent, very good, good, moderate, or poor). Spearman rank correlations are used because of non-normally distributed CPQ-G8–10 sum scores and the ordinal scaled global ratings of oral health and general well-being. It was expected that children who rated their overall health and oral health status as poor would have higher scores. According to previous studies [5, 26, 27], we expected “weak” to “moderate” (0.10–0.29 and 0.30–0.49, respectively, according to Cohen [28]) correlations between CPQ-G8–10 scores and the two global health questions, with a larger correlation with oral health than with general health.

In addition to our convergent validity analyses, we also performed (known) group validation where we determined to what degree CPQ-G8–10 scores are significantly different between groups of participants with different oral health status. Hence, we compared groups that differ regarding to two major indicators of physical oral health: the caries index (dmft/DMFT-Index; 0 versus *>* 0) and the plaque accumulation index (absent versus present). We tested CPQ-G8–10 group differences using the Mann-Whitney Wilcoxon test due to the non-normal distribution of the sum scores.

## Results

### Development of the German CPQ8–10

All 25 items of the CPQ8–10 were practicable to translate. All items of the CPQ8–10 were considered comprehensible, clear, and relevant. The children were able to answer all questions on the German instrument. When approached as to whether they had questions or needed assistance, the children indicated that they understood all questions. Therefore, no wording of the questionnaire had to be changed. No missing items were encountered.

### Study population

A total of 409 patients were initially included with mean age of 8.4 years (SD = 1.5, age range from 8 to 10 years); we used only complete observations for each analysis (column 2 in Table 1). Of these, 214 (52.3%) were girls. Fifty-eight patients completed the CPQ-G8–10 again after the test-retest time period. The mean CPQ-G8–10 sum score of all participants was 8.2 (± 9.3). In the test-retest group (58 participants), the mean CPQ-G8–10 sum score was 7.4 (±9.4) for the first assessment and 5.8 (±6.7) for the second assessment.

**Table 1:** CPQ-G8–10 score reliability in the study sample

	Number of items	Internal consistency (*N* = 340)		Test-retest reliability (*N* = 58)	
		Cronbach’s alpha (lower limit of 95% CI)	Average interitem correlation	ICC (95% CI)	Mean CPQ-G8–10 score difference examination time 1–time 2 (95% CI)
Total scale	25	0.88 (0.87)	0.51	0.85 (0.75 to 0.91)	1.54 (0.07 to 3.00)
Subscales					
Oral symptoms	5	0.58 (0.51)	0.45	0.81 (0.68 to 0.89)	0.86 (0.17 to 1.20)
Functional limitations	5	0.73 (0.69)	0.58	0.89 (0.81 to 0.93)	0.35 (−0.01 to 0.71)
Emotional well-being	5	0.81 (0.79)	0.69	0.74 (0.57 to 0.85)	0.45 (−0.14 to 1.04)
Social well-being	10	0.86 (0.83)	0.63	0.84 (0.73 to 0.90)	0.21 (−0.15 to 0.57)

### Reliability

In an examination of temporal stability in 58 patients, CPQ-G8–10 scores decreased slightly over the test-retest time period (Table 1). For the subscales, the largest change was observed for the oral symptom domain and the smallest change was observed for the social well-being domain. The ICC for the CPQ-G summary score was high (0.85 (95% CI ranging from 0.75 to 0.91)) indicating “excellent” reliability (> 0.75) according to guidelines [25]. The ICCs of the four domain subscales ranged between 0.74 and 0.89. Internal consistency (Cronbach’s alpha) for the summary score was 0.88. For the domains, the coefficient ranged between 0.58 (for oral symptoms) and 0.86 (for emotional well-being). Average interitem correlation—another measure of the scores’ internal consistency—ranged between 0.45 and 0.69.

### Validity

CPQ-G8–10 scores correlated well with other measures of the same construct (convergent validity). The scores’ correlations with global ratings of oral health and overall well-being were in the predicted magnitude (− 0.40 and − 0.14, respectively) and were statistically significant (Table 2). As we expected, the coefficient was higher for the rating of oral health than for the rating of overall well-being.

**Table 2:** CPQ-G8–10 score validity in Austrian children aged 8–10 years: correlations with self-reported oral and general health as well as caries and plaque accumulation

	***N***	Total scale (25 items)		Oral symptoms (5 items)		Functional limitations (5 items)		Emotional well-being (5 items)		Social well-being (10 items)	
		$\overline{x}$ (SD)	*r* (95% CI)	$\overline{x}$ (SD)	*r* (95% CI)	$\overline{x}$ (SD)	*r* (95% CI)	$\overline{x}$ (SD)	*r* (95% CI)	$\overline{x}$ (SD)	*r* (95% CI)
Global rating of oral health			− 0.40 (− 0.49 to − 0.31)*		− 0.31 (− 0.40 to − 0.22)*		− 0.30 (− 0.43 to − 0.25)*		− 0.36 (− 0.45 to − 0.27)*		− 0.21 (− 0.30 to − 0.11)*
Excellent	36	4.2 (6.6)		1.6 (1.6)		0.7 (1.8)		0.7 (2.0)		1.0 (2.5)	
Very good	94	5.7 (7.1)		3.2 (2.7)		1.0 (2.1)		0.8 (1.7)		0.7 (2.9)	
Good	141	7.8 (7.7)		3.5 (2.7)		1.3 (2.0)		1.5 (2.3)		1.4 (3.1)	
Moderate/poor**	69	14.5 (12.1)		5.3 (3.6)		3.2 (3.8)		3.6 (4.0)		2.1 (4.1)	
Global rating of overall well-being			− 0.26 (− 0.33 to − 0.13)*		− 0.13 (− 0.23 to − 0.03)*		− 0.26 (− 0.44 to − 0.25)*		− 0.20 (− 0.30 to – 0.10)*		− 0.14 (− 0.24 to − 0.04)*
Excellent	138	6.3 (7.7)		3.1 (2.7)		1.8 (2.1)		1.4 (2.9)		0.9 (2.2)	
Very good	129	8.7 (8.9)		3.9 (3.1)		2.1 (2.8)		1.8 (2.9)		1.5 (3.4)	
Good	68	10.4 (11.0)		4.0 (3.2)		2.2 (3.0)		2.0 (2.6)		1.8 (4.4)	
Moderate/poor**	11	16.7 (13.8)		5.0 (4.2)		4.5 (4.2)		3.7 (4.8)		2.4 (3.5)	
dmft+DMFT			Wilcoxon rank sum test ***p*** < **0.05**		Wilcoxon rank sum test n.s.		Wilcoxon rank sum test ***p*** < **0.01**		Wilcoxon rank sum test ***p*** < **0.01**		Wilcoxon rank sum test ***p*** < **0.01**
0	114	6.5 (6.8)		3.2 (2.6)		1.0 (2.0)		1.3 (2.6)		0.8 (2.0)	
> 0	232	9.0 (10.1)		3.9 (3.2)		1.9 (3.0)		1.9 (3.1)		1.5 (3.6)	
dm+DM			Wilcoxon rank sum test ***p*** < **0.01**		Wilcoxon rank sum test n.s.		Wilcoxon rank sum test ***p*** < **0.001**		Wilcoxon rank sum test ***p*** < **0.01**		Wilcoxon rank sum test ***p*** **= 0.00013 p** < **0.01**
0	153	6.4 (6.7)		3.2 (2.6)		1.0 (1.9)		1.4 (2.7)		0.8 (2.2)	
> 0	193	9.7 (10.6)		4.0 (3.3)		2.1 (3.1)		2.0 (3.1)		1.7 (3.7)	
Plaque			Wilcoxon rank sum test n.s.		Wilcoxon rank sum test n.s.		Wilcoxon rank sum test ***p*** **= 0.05**		Wilcoxon rank sum test ***p*** < **0.01**		Wilcoxon rank sum test *p* = 0.3981 n.s.
Absent	167	7.5 (8.6)		3.4 (2.8)		1.3 (2.2)		1.5 (2.7)		1.3 (3.3)	
Present	157	9.1 (10.1)		3.8 (3.2)		1.9 (3.2)		2.2 (3.3)		1.4 (3.3)	

**Categories moderate and poor were combined because very few children rated their oral health as “poor”; *p < 0.05; Wilcoxon rank sum tests were performed to take into account the right skewed, non-normally distributed data; significant *p* values are marked in bold

When patients were grouped according to health indicators as caries and plaque accumulation, differences in CPQ-G8–10 scores were detected. Results of the assessment of discriminant validity indicated that overall, children with ≥ 0 decayed and/or treated teeth/surfaces had higher CPQ-G scores than those who were caries-free. As predicted, differences in children with and without plaque accumulation were also present and statistically significant (Table 2).

## Discussion

This study adapted the original English language CPQ8–10 version to the German language and investigated its psychometric properties in Austrian children aged 8–10 years. The translation and cross-cultural adaptation were carefully conducted following the four-step procedure recommended by Beaton et al. [17]. This process resulted in a back-translated version (CPQ-G8–10) that was very similar to the original. We found that all of the questions could be used in the German context. CPQ-G8–10 scores were reliable and valid in the general population.

Our results from the reliability assessment were comparable with findings from previous studies. For the evaluation of test-retest reliability, the time interval between the administrations of the questionnaires was restricted to 3 weeks in the current study, which was in agreement with previous studies that used a time interval of 2 to 3 weeks [7, 13, 14]. The test-retest reliability sample showed stability in responses to the German version of the CPQ8–10. The ICC for the total scale was 0.85, indicating excellent reproducibility according to guidelines. In a study examining the Brazilian version, the ICC was slightly higher (0.96) [13]. However, in the original version, the ICC for the overall scale was lower (0.75) [7].

In relation to internal consistency, Cronbach’s alpha was high for the total score. Within the subscales, the score ranged between 0.58 for oral symptoms and 0.86 for social well-being indicating adequate internal reliability, as reliability of 0.5 or above is considered acceptable [20, 25]. Our internal consistency results were similar to findings in the original English version (*α* = 0.89) [7], the Brazilian and the Cambodian version (*α* = 0.88) [13, 15], and the Danish version (*α* = 0.87) [11]. All these versions showed overall high internal consistency with the oral symptom scale presenting a lower score, ranging between 0.57 and 0.67 [7, 11, 13, 15].

Global ratings of oral health and overall well-being have been used to validate instruments for OHRQoL in other studies [7, 11, 13, 15]. Construct validity of the German CPQ-8–10 was as predicted. The correlations between both global ratings and the summary score were of moderate magnitude, statistically significant, and followed predicted patterns. There was a score gradient by global item response, and those who responded with “Moderate/Poor” had significantly higher scores than those in the less severe response categories. These findings were in agreement with the majority of previous CPQ8–10 questionnaire studies. In a study examining the French version of the CPQ for 8- to 10-year-olds, significant correlations were also seen between global ratings of oral health or overall well-being and the summary score and all subscales [10]. Moreover, the French as well as the Brazilian version reported similar correlation scores for the global rating of oral health (0.38). In the original version, Jokovic et al. also observed significant correlations between all subscale scores and both global ratings, except between the functional limitations and social well-being scores and oral health rating [7].

Furthermore, we determined discriminant validity by comparing CPQ-G8–10 scores between children with and without caries experience, and plaque accumulation being absent or present, respectively. We hypothesized that 8–10-year-old children with caries would have higher OHRQoL impacts. Our findings showed that children with caries (dmft+DMFT > 0) did indeed have significant higher scores than those who did not present caries. However, variations in the mean CPQ and the four subscale scores were apparent. Higher scores were found in all four subscales and the total scale among those children with at least one carious lesion compared with those who were caries-free, indicating worse OHRQOL. This is consistent with the original English version of the questionnaire [7]. Jokovic et al. reported that caries-free children reported significant lower scores in the overall scale and in each domain, compared with children with decayed teeth. Likewise, they found the mean social well-being score being two times higher in caries-affected group. Moreover, results of the Brazilian study point into the same direction, providing evidence to suggest that the CPQ8–10 scores were associated with the severity of caries being present or absent [13]. In addition, Aguilar-Diaz et al. who validated the Spanish version of the CPQ8–10 in use with Mexican urban children also detected that children who did not present caries (dmfs and DMFS = 0) tended to report lower scores in the overall scale and in each domain, compared with children who have had caries experience, whether they had cavities, or filled or lost teeth [14]. When we investigated the relationship of CPQ-G8–10 scores to plaque indicators, the findings pointed into a comparable direction. Children with plaque being present showed higher CPQ8–10 sum scores than those with plaque being absent. Although these differences were not statistically significant (except for emotional well-being and functional limitations), our results are comparable with those for German children aged 11 to 14 years. Bekes et al. found similar results in their study when focusing on plaque accumulation [1].

As other oral pathologies might be present in this age group, additional research should be considered regarding the discriminant validity of this version in studying clinical conditions, such as malocclusion or molar incisor hypomineralization, the latter being a growing issue.

## Conclusion

The German version of the CPQ questionnaire for 8- to 10-year-old children is a valid and reliable instrument to measure OHRQoL. The use of this instrument can help to describe the impact of dental diseases (e.g., caries) and their treatment on children in this age group. Furthermore, it provides the opportunity to compare similarities and differences in oral health impacts among this age group in different countries.

## Electronic supplementary material

ESM 1 (DOCX 24 kb)
