# Supplementary material for: The German version of Child Perceptions Questionnaire for children aged 8 to 10 years (CPQ-G8–10): translation, reliability, and validity
Source: Clin Oral Investig. 2020 Jul 14;25(3):1433–9. doi: 10.1007/s00784-020-03451-w (PMC7878203; doi:10.1007/s00784-020-03451-w)
Supplement: Supplementary file 1 — (DOCX 24 kb) [file 784_2020_3451_MOESM1_ESM.docx]

**Fragebogen zur Mundgesundheit**

*Hallo,*

*danke, dass Du uns bei unserer Studie hilfst!*

*Wir führen diese Studie durch, um besser die Dinge zu verstehen, die Kindern aufgrund ihrer Zähne und des Mundes passieren.*

*Bitte beachte:*

- *Schreibe Deinen Namen nicht auf den Fragebogen.*
- *Dies ist kein Test und es gibt keine richtigen oder falschen Antworten.*
- *Antworte so ehrlich wie Du kannst.*
- *Sprich mit keinem über die Fragen, wenn Du sie beantwortest.*
- *Keiner, den Du kennst, wird die Antworten sehen.*
- *Lies jede Frage sorgfältig durch und denke an die Dinge, die Dir in den letzten 4 Wochen passiert sind.*
- *Bevor Du antwortest, frage Dich selber: “Passiert mir dies wegen meiner Zähne oder wegen meines Mundes?”*
- *Kreuze die Antwort an, die am besten passt.*

**Fragebogen zur Mundgesundheit**

**ZUERST EIN PAAR FRAGEN ÜBER DICH**

Heutiges Datum: _________________

Bist Du ein Junge/Bub oder ein Mädchen? Junge Mädchen

Wie alt bist Du? _________________

|  | ***Ausge-zeichnet*** | ***Sehr gut*** | ***Gut*** | ***Mittel-mäßig*** | ***Schlecht*** |
| --- | --- | --- | --- | --- | --- |
| Wie würdest Du Deinen allgemeinen Gesundheitszustand einschätzen? |  |  |  |  |  |
| Wie würdest Du Deinen Mundgesundheitszustand einschätzen? |  |  |  |  |  |

**NUN EIN PAAR FRAGEN ZU DEINEN ZÄHNEN UND DEINEM MUND**

|  | ***Nie*** | ***1x oder 2x*** | ***Gele-gent-lich*** | ***Oft*** | ***Sehr oft*** |
| --- | --- | --- | --- | --- | --- |
| **Wie oft hast Du in den letzten vier Wochen...?** | | | | | |
| Schmerzen an Deinen Zähnen oder im Mund gehabt |  |  |  |  |  |
| wunde Stellen in Deinem Mund gehabt |  |  |  |  |  |
| Schmerzen an Deinen Zähnen beim Trinken kalter Getränke oder beim Essen von Speisen gehabt |  |  |  |  |  |
| schlechten Atem gehabt |  |  |  |  |  |
| **Wie oft ist in den letzten vier Wochen ...?** | | | | | |
| Essen an den Zähnen hängen geblieben |  |  |  |  |  |
| **Wie oft hast Du aufgrund Deiner Zähne oder Deines Mundes in den letzten vier Wochen ...?** | | | | | |
| länger gebraucht als andere, um Deine Mahlzeit zu essen |  |  |  |  |  |
| Schwierigkeiten beim Essen oder Kauen von Speisen wie Äpfel, Maiskolben oder Fleisch gehabt |  |  |  |  |  |
| Schwierigkeiten beim Essen von Speisen, die Du gerne essen möchtest, gehabt |  |  |  |  |  |
| Schwierigkeiten beim Sprechen von einigen Wörtern gehabt |  |  |  |  |  |
| ein Problem beim Schlafen in der Nacht gehabt |  |  |  |  |  |

**EINIGE FRAGEN ÜBER DEINE GEFÜHLE**

|  | ***Nie*** | ***1x oder 2x*** | ***Gele-gent-lich*** | ***Oft*** | ***Sehr oft*** |
| --- | --- | --- | --- | --- | --- |
| **Wie oft hast Du Dich wegen Deiner Zähne oder Deines Mundes in den letzten vier Wochen... ?** | | | | | |
| geärgert |  |  |  |  |  |

|  | ***Nie*** | ***1x oder 2x*** | ***Gele-gent-lich*** | ***Oft*** | ***Sehr oft*** |
| --- | --- | --- | --- | --- | --- |
| **Wie oft warst Du wegen Deiner Zähne oder Deines Mundes in den letzten vier Wochen ... ?** | | | | | |
| frustriert |  |  |  |  |  |
| schüchtern |  |  |  |  |  |
| betroffen, was andere Leute denken |  |  |  |  |  |
| besorgt, dass Du nicht so gut aussiehst |  |  |  |  |  |

**FRAGEN ZU DEINER SCHULE**

|  | ***Nie*** | ***1x oder 2x*** | ***Gele-gent-lich*** | ***Oft*** | ***Sehr oft*** |
| --- | --- | --- | --- | --- | --- |
| **Wie oft hast Du wegen Deiner Zähne oder Deines Mundes in den letzten vier Wochen ...?** | | | | | |
| in der Schule gefehlt |  |  |  |  |  |
| Schwierigkeiten beim Erledigen von Hausaufgaben gehabt |  |  |  |  |  |
| Schwierigkeiten gehabt, in der Schule aufzupassen |  |  |  |  |  |
| nicht laut sprechen oder lesen wollen |  |  |  |  |  |

**FRAGEN ZUM ZUSAMMENSEIN MIT ANDEREN MENSCHEN**

|  | ***Nie*** | ***1x oder 2x*** | ***Gele-gent-lich*** | ***Oft*** | ***Sehr oft*** |
| --- | --- | --- | --- | --- | --- |
| **Wie oft hast Du wegen Deiner Zähne oder Deines Mundes in den letzten vier Wochen ... ?** | | | | | |
| versucht nicht zu lächeln oder zu lachen im Beisein anderer Kinder |  |  |  |  |  |
| nicht mit anderen Kindern sprechen wollen |  |  |  |  |  |
| nicht mit anderen Kindern zusammen sein wollen |  |  |  |  |  |
| **Wie oft bist Du wegen Deiner Zähne oder Deines Mundes in den letzten vier Wochen ...?** | | | | | |
| weggeblieben von Akitivitäten wie Sport oder Vereinen |  |  |  |  |  |
| von anderen Kindern gehänselt oder beschimpft worden |  |  |  |  |  |
| **Wie oft haben wegen Deiner Zähne oder Deines Mundes in den letzten vier Wochen ...?** | | | | | |
| andere Kinder Fragen gestellt |  |  |  |  |  |
